# Influence of substrate modulus on gecko adhesion

**DOI:** 10.1038/srep43647

**Published:** 2017-03-13

**Authors:** Mena R. Klittich, Michael C. Wilson, Craig Bernard, Rochelle M. Rodrigo, Austin J. Keith, Peter H. Niewiarowski, Ali Dhinojwala

**Affiliations:** 1 University of Akron, Department of Polymer Science, Akron, OH, 44235 USA; 2 University of Akron, Department of Biology, Akron, OH, 44235 USA; 3 University of Akron, Program in Integrated Bioscience, Akron, OH, 44235 USA

## Abstract

The gecko adhesion system fascinates biologists and materials scientists alike for its strong, reversible, glue-free, dry adhesion. Understanding the adhesion system’s performance on various surfaces can give clues as to gecko behaviour, as well as towards designing synthetic adhesive mimics. Geckos encounter a variety of surfaces in their natural habitats; tropical geckos, such as *Gekko gecko*, encounter hard, rough tree trunks as well as soft, flexible leaves. While gecko adhesion on hard surfaces has been extensively studied, little work has been done on soft surfaces. Here, we investigate for the first time the influence of macroscale and nanoscale substrate modulus on whole animal adhesion on two different substrates (cellulose acetate and polydimethylsiloxane) in air and find that across 5 orders of magnitude in macroscale modulus, there is no change in adhesion. On the nanoscale, however, gecko adhesion is shown to depend on substrate modulus. This suggests that low surface-layer modulus may inhibit the gecko adhesion system, independent of other influencing factors such as macroscale composite modulus and surface energy. Understanding the limits of gecko adhesion is vital for clarifying adhesive mechanisms and in the design of synthetic adhesives for soft substrates (including for biomedical applications and wearable electronics).

Geckos have been intriguing for both biologists and engineers due to their dry adhesive system, which allows them to stick to smooth and rough surfaces^1^,^2^,^3^,^4^, as well as underwater^5^,^6^,^7^,^8^,^9^. Their adhesion system is based on making intimate contact with a surface over a large area, utilizing van der Waals forces as their main adhesion mechanism^10^,^11^. This large contact area is obtained through a hierarchical structure on the geckos toes. The toes are striped with lamellae, each of which has long beta-keratin hairs, or setae, which then subdivide into branches; on the tips of these branches are spatulae, plate-like structures ∼200 nm across at their widest point. These spatulae ultimately contact the surface. For adhesion to occur, the spatulae must be aligned with the surface through a shearing motion. This pulls the spatulae from a non-adhesive state to an aligned adhesive state^12^. The oriented spatulae intimately contact the surface, allowing van der Waals adhesion to occur. Once the peeling angle reaches 30°, the setae release from the surface, terminating adhesion^13^.

We continue to learn more about adhesive design from the performance of geckos in challenging adhesion conditions. In nature, geckos face rough, flexible, dirty surfaces on a regular basis^14^. Considerable work has been done on hard surfaces to elucidate the effect of different surface chemistries^6^,^7^,^8^,^9^, roughnesses^2^,^4^,^14^,^15^,^16^, friction coefficients^17^, and electrostatic interactions on gecko adhesion^18^. In parallel, the development of many synthetic adhesives has been inspired by the gecko adhesion system. An attractive area for the use of these adhesives is in the biomedical field. A balance between good adhesion to tissue and easy, non-damaging removal is a key challenge for biomedical adhesives. The structured adhesive system, inspired by geckos, is appealing due to its lauded adhesion, compliance to rough surfaces, and clean removal. Biomedical structured synthetics, therefore, have been made and tested specifically for soft substrates, an area in which there have been no targeted gecko studies. Both unstructured and structured synthetic adhesives have been designed for use on skin and biological tissue^19^,^20^,^21^. Biological tissues are challenging substrates, as they typically exhibit both roughness and softness, in addition to operating in wet environments. Structural adhesives have been designed for these challenges using a variety of strategies, including adding chemically-bonding components to the adhesive surface^22^. While this strategy works well for underwater adhesion, for re-usable, easy-peel, structured adhesives permanent bonding is not an option. Other strategies include tailoring the pillar aspect ratios and elastic modulus. Increasing the pillar aspect ratio improves pillar adhesion to skin over a flat, unstructured adhesive; however, if the aspect ratio is too high, bundling occurs^20^. To improve contact with the surface, the entire pillar’s modulus can be reduced^19^. Alternatively, a high modulus pillar can be terminated with a low modulus tip, allowing for overall strength while maintaining high surface contact^20^.

The synthetic pillar studies on skin only provide a partial picture, as the modulus shifts of the pillars are relatively limited in scope, due to pillar bundling at low modulus. When the substrate itself is varied in modulus, synthetics have shown a shift in adhesion. A study of large, synthetic, mushroom-tipped structural adhesives on soft F-15 polyurethane substrates (E∼200 kPa) of various thicknesses supported by glass showed a drop in normal adhesion with increasing layer thickness, leveling off after the 1.5 mm thick substrate. The lowest measured force of adhesion for these mushroom pillars was 2 N/cm^2^, a drop of 5x over the 0.02–5.5 mm thick polyurethane sheets^23^. While modulus was not explicitly measured, it should shift with thickness, indicating that for mushroom-tipped pillars there is a positive correlation of normal adhesion with substrate modulus. These pillars are quite different from the gecko adhesion mechanism and morphology, but there could be a similar effect of modulus on gecko adhesion.

Although there have been no direct studies of the influence of substrate softness on gecko adhesion, there have been studies where softness may have played an unexplored underlying role. Two previous animal studies have included polydimethylsiloxane (PDMS) substrates. A soft elastomer, PDMS has a Young’s modulus of ∼2 MPa^24^. While both studies were designed to investigate other aspects of gecko adhesion, the use of PDMS as a substrate introduced the complicating factor of softness. When tested in a vertical orientation, two gecko feet were found to have an adhesion of 1 N/cm^2^ on PDMS^18^. In whole animal horizontal testing, geckos have been shown to have limited shear adhesion to a thick, soft layer of PDMS in air (on average, less than 0.3 N/cm^2^)^9^. It was unclear why the geckos stuck so poorly to this smooth hydrophobic substrate in air, when other similar hard hydrophobic substrates (such as glass coated with an octadecyltrichlorosilane self-assembled monolayer (OTS-SAM)) have not inhibited their shear adhesion^6^. Given that geckos adhere to a wide variety of natural materials, including leaves (some of which have elastic moduli in the MPa range^25^), it is surprising that softness could be a limiting factor.

Here, we have investigated for the first time the impact of softness on gecko adhesion using both whole animal and single lamella experiments. Softness is a broad term, and it is unclear what ‘soft’ means for gecko adhesion. We have defined ‘softness’ here in terms of a substrate’s modulus. Gecko adhesion is highly hierarchical, depending not only on the whole animal, but also on the nanometer thin spatulae that contact the surface. As we do not know which length scale is critical for adhesion on soft substrates, we have defined two different scales of modulus: one on the macroscale, which is the modulus of the whole substrate assembly (referred to here as composite modulus) and one on the nanoscale, which is the modulus of the surface layer of the substrate assembly (surface modulus). We measured the whole animal shear adhesion of *Gekko gecko* on two different substrates: cellulose acetate and PDMS. Within each surface type, composite modulus was varied across 5 orders of magnitude. This allowed us to determine whether gecko adhesion is affected by soft, low-modulus substrates, and over what scale modulus influences shear adhesion. Understanding this relationship has implications for understanding gecko’s adhesion on more complex, biologically relevant substrates, as well as for future experimental design considerations.

## Results

### Surface Properties

Both cellulose acetate and PDMS substrates were made with different composite moduli (see Methods for details). For the cellulose acetate substrates, this involved varying the underlying layer between the cellulose acetate sheet and the supporting glass. For the PDMS substrates, the thickness of the PDMS layer was varied. The cellulose acetate composites’ moduli were calculated from hardness measurements made following ASTM D 2240-04. Hardness measurements were done with a Shore A durometer, which requires a minimum substrate thickness of 0.25 inches. Most of the PDMS substrates were too thin to have accurate readings using this technique, so their effective moduli were calculated using a simple parallel spring model, as outlined in the Methods section. The calculated effective moduli of the substrates, along with their contact angles, are presented in Table 1.

If the modulus of the interface between a spatula and the substrate is critical for gecko adhesion, then characterizing the composite modulus is inadequate. The surface modulus of each substrate was measured using a nanoindenter, which uses micron sized tips to indent a material with a given force. The distance the tip can travel (≤1 μm) with the given force can be related to the modulus of the surface layer of the material (provided in Table 1). The surface layer modulus is influenced both by the modulus of the surface material, as well as that of the underlying layers (although this substrate effect diminishes with increasing distance from the surface). The nanoindenter results for the 10 μm and the 1000 μm PDMS substrate differ by only 2 MPa, whereas the composite moduli for the same substrates differ by nearly an order of magnitude.

A concern with the use of PDMS for the low modulus materials is the potential formation of a lubricating layer of oligomers on the surface, should the material not have completely crosslinked. The PDMS used was not solvent extracted prior to testing, as solvent extraction removed the PDMS sheet from the supporting glass. Therefore, there could be a slippery surface formed on the PDMS substrates. As the 2 nm PDMS layer was adsorbed onto the glass plate, rather than cast, the free oligomers were minimized for the high modulus sample. The influence of the potential oligomer layer on shear adhesion was investigated by measuring the friction coefficients of the PDMS substrates (Table 2), where the friction coefficient is the ratio of the shear force to the normal force. It is unknown whether a stiff lens or a flexible lens would be the best model for the gecko system; here, we have used the flexible PDMS lens, leading to high coefficients of friction (from literature, PDMS/PDMS has a coefficient of friction of ∼2^26^, compared to the coefficient of friction for a human finger against glass, which is ∼0.6^27^). The friction of the pristine unextracted PDMS was compared to the extracted PDMS; while there is a difference in the friction coefficients of the two substrates, the friction coefficient of the unextracted PDMS is comparable to clean glass measured under the same conditions, to which geckos have been demonstrated to stick well^6^. Therefore, any presence of an oligomeric lubrication layer should not substantially affect the ability of the geckos to stick to the PDMS surface.

### Shear Adhesion Measurements

#### Whole Animal Experiments

**Figure 1:**
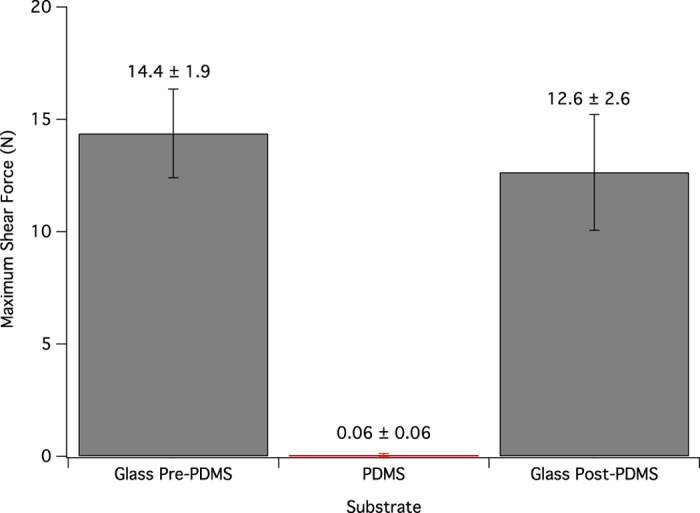
Average maximum shear force for whole animals on glass pre-exposure to PDMS, to the PDMS surface, and finally to the glass surface after testing on PDMS. Values are means ± standard error, n = 7. The difference in shear adhesion values on glass pre and post exposure to PDMS is not statistically significant.

While the potential lubrication layer present on the PDMS samples should not affect the gecko’s ability to adhere, there is the possibility of toe contamination. To address this concern, gecko shear adhesion on glass was compared before and after a trial on PDMS; had the oligomers transferred to the gecko’s toes, the shear adhesion post PDMS should have decreased. There was no significant difference (p = 0.5651, Table 3) in shear adhesion before and after PDMS trials (Fig. 1). Because the difference was insignificant compared to the random error of the shear adhesion experiment, the effect of toe contamination is considered to be negligible on shear adhesion across multiple trials.

**Figure 2:**
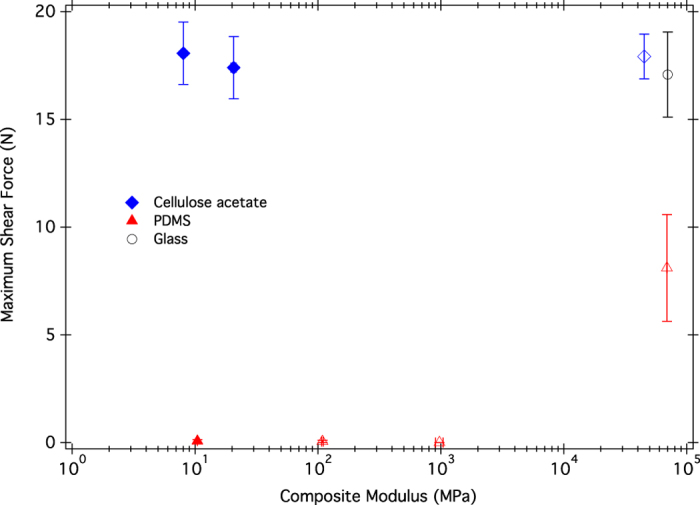
Average maximum shear force of adhesion for whole animals on cellulose acetate (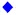) and PDMS (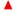) substrates of varying composite moduli. Composite moduli are calculated from the spring model (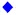, 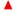) or from durometer measurements (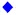, 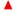) and are tabulated in Table 1. Values are means ± standard error, n = 7.

**Figure 3:**
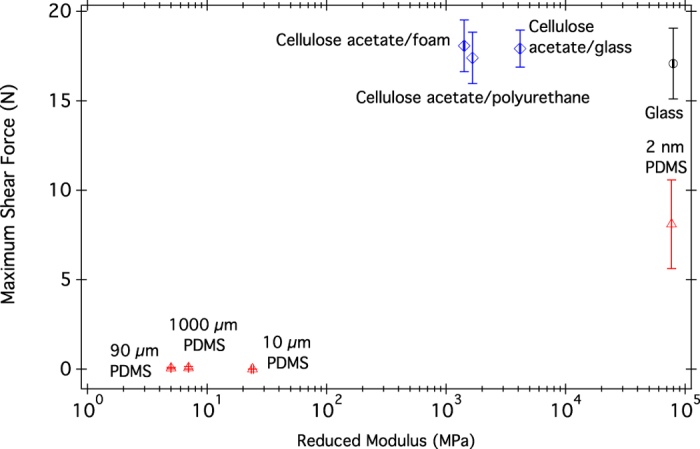
Average maximum shear force of adhesion for whole animals on cellulose acetate and PDMS substrates as a function of the surface modulus of the substrates (as measured using a nanoindenter). Individual points are labeled, and the thicknesses of the surface layer are tabulated in Table 1. Values are means ± standard error, n = 7.

**Figure 4:**
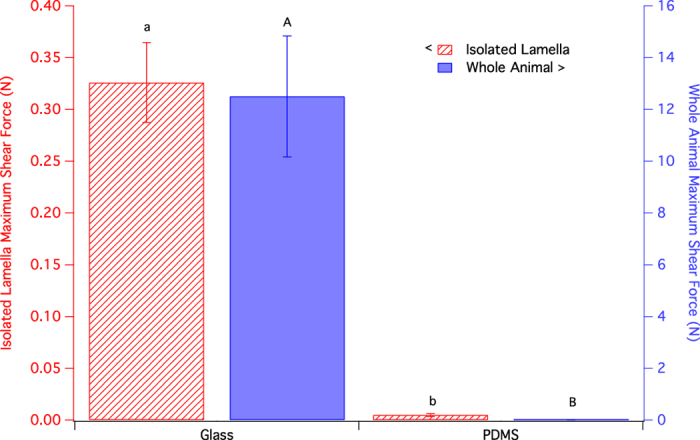
Average maximum shear force of adhesion for whole animal and isolated lamella on glass and 1000 μm PDMS substrates. Values are means ± standard error, n = 7 for both data sets. Results of student’s t-test are labeled in lower case for the isolated lamella experiments, and in upper case for the whole animal experiments.

As seen in Figs 2 and 3, the maximum shear force of gecko shear adhesion on the two sets of substrates varied considerably. Strong shear adhesion was seen on the cellulose acetate substrates(∼17 N for all cellulose acetate substrates). On PDMS substrates with composite moduli <GPa, however, the geckos were unable to support their own weight. The single PDMS substrate to which the geckos adhered successfully was the 2 nm PDMS coated glass (69 ± 0.2 GPa composite modulus). A repeated measures ANOVA was run comparing the shear force of whole animals on glass and 1000 μm PDMS, followed by a student’s t-test. Gecko maximum shear force was dependent on the substrate (Table 3), and geckos had different shear adhesion on each substrate (marked A and B in Fig. 4).

#### Isolated Lamella Experiments

Given that the geckos showed a distinct aversion for the PDMS substrates, the influence of gecko behaviour on our shear adhesion measurements was also a concern. To ensure that the gecko’s lack of adhesion to the PDMS substrates was not due to this behaviour, single lamellae were removed from naturally molted skin and mounted on glass substrates. Testing these lamellae in isolation of the animal, however, changes the environment of the lamella. On the animal, a lamella is part of an assembly, supported by skin and tendons. The compliance of this assembly has been shown to be a component of gecko adhesion^21^,^28^,^29^. We did not measure the compliance of the animal, but others have modeled the whole animal compliance as a series of springs (setae, skin, tendons, and bones)^28^,^29^,^30^,^31^. We assume that the replacement of this series of springs with our glass support decreases the system compliance. This difference in system compliance between the isolated lamella samples and the whole animal means that the values from isolated lamella experiements cannot be scaled or directly compared. Instead, we have examined the gecko system’s adhesion to a thick PDMS surface within each experimental set-up.

The adhesion of isolated lamellae was tested on the 1000 μm PDMS substrate, with the glass substrate as a control. The results are plotted in Fig. 4, along with the performance of the whole animals on the same substrates as a reference. The shear adhesion depended on the substrate (see Table 3), with a p-value of 0.0002. The average shear force of the isolated lamellae on glass was 0.33 ± 0.04 N, while on PDMS, the average shear force was 0.005 ± 0.001 N. The isolated lamella adhered well to glass, indicating that the sample preparation methods did not inhibit the lamella’s ability to stick. However, shear adhesion of isolated lamella on PDMS was 66x lower than that of the isolated lamella on glass. This indicates that the gecko adhesion system, even in a controlled set up in isolation of the animal, cannot stick to the 1000 μm PDMS.

## Discussion

The testing of geckos on varying modulus substrates revealed several surprises. Firstly, there was no change in shear adhesion with varying composite modulus for the cellulose acetate substrates, indicating that gecko shear adhesion was not affected by underlying soft layers. Secondly, on the low modulus surfaces of PDMS, geckos could not support their own weight. This is contrary to expectations of improved contact between deformable substrates. As geckos primarily adhere using van der Waals forces^10^, shear adhesion is dependent on surface contact, a parameter that typically increases on soft substrates. This low shear adhesion on PDMS could have several possible contributing components: surface chemistry, capillary adhesion, friction, composite modulus, and surface modulus. Geckos have been shown to stick to both cellulose acetate^32^ and PDMS^18^, however PDMS has been shown to be a more challenging substrate^9^. From our 2 nm PDMS substrate, which has the modulus of glass but the surface chemistry of PDMS, it is evident that geckos can stick to PDMS. This is expected for van der Waals adhesion, which depends on the polarizability of the material^10^, which should not vary greatly between the cellulose acetate sheet and the PDMS.

The cellulose acetate surface is hydrophilic, while the PDMS is hydrophobic (contact angles in Table 1), so there could be capillary contributions to the shear adhesion on cellulose acetate substrates. Tests were run at 25 °C, 35 ±5% RH to decrease the vapor pressure and minimize condensation. At 35% RH, the normal pull-off force for a single spatula on hydrophilic glass is ∼9 nN, while on hydrophobic OTS coated silicon wafers, the normal pull-off force was slightly lower, ∼7 nN^33^. Capillary forces may contribute to the higher shear forces seen in our measurements on hydrophilic cellulose acetate compared to the hydrophobic PDMS, but they are not the sole source of adhesion. The drop in shear adhesion seen from the 2 nm PDMS substrate to the 10 μm PDMS substrate could not be due to a change in capillary adhesion, as both substrates were PDMS. Changes in the force of capillary based adhesion could be expected for changes in surface chemistry, which would affect the contact angle of the water bridge connecting the two materials. Since the 2 nm PDMS substrate and the 10 μm PDMS substrates have similar water contact angles, it would not be expected for there to be changes in any capillary adhesion.

Reduced friction on the cast PDMS substrates could contribute to the low shear adhesion on PDMS, as the presence of a lubrication layer on the PDMS is likely. The friction coefficient is lower for the pristine PDMS than the extracted PDMS, but the friction coefficients are still within the range of materials to which geckos have previously adhered well^6^. Therefore, the lack of shear adhesion to the cast PDMS surfaces, yet successful adhesion to the thin 2 nm adsorbed PDMS substrate, cannot be simply due to surface chemistry, capillary forces, or friction.

The last main differences between the surfaces are their composite and surface moduli. There is no shift in shear adhesion across composite modulus for the cellulose acetate samples (Fig. 2). The surface moduli of the cellulose acetate substrates are the same for all samples, and are higher than the surface moduli of the 10, 90, and 1000 μm PDMS substrates. Across the PDMS substrates, there is no shift in shear adhesion until the composite modulus is >GPa. However, it is important to note that for the >GPa PDMS substrate, the surface coating is so thin that the surface modulus is also high (77 GPa, as measured by nanoindenter). The 90 and 1000 μm samples have the same surface moduli: 5 and 7 MPa respectively. Across all substrates tested, there is no correlation with composite modulus, but there is a correlation of shear adhesion with the surface modulus (Fig. 3). At a low surface modulus, there is no shear adhesion.

Clearly the surface layer modulus is interacting with the gecko hierarchical adhesion system. However, we have no direct evidence as to which length scale of the gecko system determines adhesion to this surface layer. On the whole animal scale, there is the behaviour of the animals themselves. We noticed that the geckos avoided the low surface modulus PDMS substrates when possible; it is unclear whether this was due to them searching for a better hold, or if there was a sensory component. Such behaviour led to mistrials, and did not contribute to the presented data. Our concerns that this aversion to PDMS was influencing our trials were addressed through the isolated lamella experiments. Isolated lamellae’s poor adhesion to the 1000 μm PDMS substrate support our whole animal results, indicating that the poor adhesion on low surface modulus substrates was not a result of gecko behaviour. While the isolated lamella experiments were used to clarify this point, we caution against drawing conclusions from force scaling of the lamella to estimate whole animal adhesion. There remains a disparity between forces measured by single seta measurements and those measured in whole animal^13^. We do not fully understand the interplay between parts of the gecko’s hierarchical structure, nor the role of the supporting structure of the animal. The whole animal and isolated lamella experiments differ not only in the removal of gecko behaviour, but also in their system compliance. This decrease in compliance in comparison with the whole animal likely influences the magnitude of adhesion. Given these differences between the gecko samples tested in isolated lamella and whole animal experiments, the similarity between their adhesion trends may indicate that the poor adhesion on 1000 μm PDMS is dominated by the seta/spatula assembly interacting with the surface modulus of the sample.

As the setae and spatulae encounter the low surface modulus of the thick PDMS samples, the surface may deform around the contact. Typically, deformation increases adhesion, as deformation of the substrate increases the contact area of a probe with the surface. However, due to the gecko adhesion’s dependence on alignment of the setae and spatulae with the surface, deformation may act as a hinderance to adhesion. If the setae are restricted from aligning by this deformation of the surface, the ability of the gecko to engage the setae and spatulae through shear alignment could be diminished. If the surface deforms at an angle with the seta that is higher than 30°, the seta will detach^13^. This angle of detachment was found to be lower for setal arrays and individual gecko toes, both of which detached at ∼25° 17. If the setae are instantly detached, the shear adhesion is then solely reliant on friction, rather than van der Waals interactions. Friction measurements of both aligned and unaligned setal arrays have been measured on glass; when isolated setal arrays were sheared in the opposite direction of normal attachment, their average shear force was ∼7.5 mN. This was 10x lower than the shear force of setal arrays sheared in the natural direction (∼75 mN), where the setae and spatula were able to align and adhere to the glass substrate^17^. If the setae are not aligned with the substrate, the geckos may not be able to support their body weight on the PDMS substrates. On the higher modulus substrates, it may be easier for the setae to contact the surface, as the substrates would deform less.

If the setae are not instantly detached, the gecko system must still align the spatulae to achieve adhesion. If a single seta has been preloaded, but not engaged, the normal force has been shown to be <1 μN, a drop of 96% compared to when the spatulae have been pulled into alignment^13^. On the 2 nm PDMS substrates, where the geckos were engaging, they supported a force of 8 ± 2.5 N. If we assume similar behaviour for engaged spatulae on the thicker PDMS substrate, and that shear force drops proportionally to the normal force, then a drop of 96% in shear adhesion for whole animals on the 2 nm PDMS substrate would be ∼0.32 N, a force insufficient to support their body weight. Our geckos struggled to support their own weight on the thick PDMS substrates, sliding down and resulting in measurements of 0 N shear force. This could be a result of the spatulae not engaging on the low modulus surfaces of the 10, 90, and 1000 μm PDMS.

Finally, the poor shear adhesion of the geckos to the soft surfaces could be related to the physics of the spatula adhering and shearing on a soft surface, rather than any lack of contact. Modeling this adhesion is not trivial, as shear adhesion on these soft substrates is geometry dependent. Measurements have been done with a synthetic gecko mimic; the mushroom shaped pillars were brought into contact with a series of soft substrates with different thicknesses and pulled off in a normal direction. It was shown that the pillar adhesion was a function of substrate thickness, increasing as the soft substrate decreased in thickness^23^. Similar results were seen with a punch pulled normally off of a soft substrate, again varying in thickness. As thickness decreased, the critical pull-off stress increased^34^. Both of these studies linked the increase in adhesion with the different stress concentrations that developed between the pillar/stub and the surface. For a thick, soft substrate, the stress concentrations at the edge of the pillar or stub were found to initiate on the edge, propagating quickly across the interface. With a thin, soft substrate, the surface could not readily deform at the edges of the pillar/stub, and the surface instead separated from the center of the stub, resulting in cavitation, which corresponded to a higher total critical pull-off stress. While these trends parallel the shear adhesion results seen in the gecko system on soft substrates, the geometries are very different. In the gecko system, forces are likely applied to the triangular spatula in both shear and normal directions, resulting in different stress distributions. We cannot directly link the shear adhesion of this hierarchical fibrillar structure with the normal adhesion of the pillars or stubs on soft substrates.

We have observed that geckos can stick to a thin adsorbed layer of PDMS, but are unable to stick to PDMS substrates that are 10 μm, 90 μm, and 1000 μm thick, while they stick to cellulose acetate surfaces with similar composite moduli. Possible reasons for this difference in shear adhesion are the different surface chemistries of the substrates, resulting in different capillary adhesion, the potentially low friction of PDMS surfaces, and the substrates’ moduli. We have eliminated the influence of surface chemistry and capillary adhesion, the friction coefficient of PDMS, and the composite modulus. It remains to be determined how the setae and spatulae are interacting with the surfaces of these low moduli substrates, and where the line between adhesion and failure lies. A careful study on the specific nanoscale geometry of the spatula/low modulus material contact point is required to clarify this structural interaction. The true contact between spatulae and testing surfaces during shear adhesion and peeling has yet to be visualized, therefore the current models remain hypotheses. These models do not allow us to differentiate between the possible sources of the gecko’s adhesive failure on soft surfaces. It is clear that the geckos are insensitive to moduli of the substrates on a large length scale, but that the modulus of the layer in contact with the setae and spatulae has a critical effect on gecko shear adhesion. This effect was seen on both the whole animal scale and on the isolated lamella scale. ‘Soft’ for a gecko, therefore, is determined by the surface layer only. Many substrates colloquially considered soft (such as leaves) may be indistinguishable from ‘hard’ substrates for geckos. Future work with relevant natural materials would be a new avenue for understanding geckos interactions with their environment. While the poor ability of geckos to stick on soft substrates has been mentioned anecdotally^35^, this is the first study to investigate the role of a substrate’s modulus on gecko adhesion. As the field of gecko adhesion forges forward into more complex and natural surfaces, we urge careful consideration of the materials used. Understanding the limits of gecko adhesion is vital both for clarifying adhesive mechanisms and for synthetic adhesive design, particularly those intended for use as biomedical patches or wearable electronics.

## Methods

### Surface Preparation

The control glass substrate was thoroughly cleaned (soap/water, acetone, methanol, base bath, DI water, nitrogen dried) to remove any contaminants, then sheeted with water and ethanol repeatedly, to mimic the cleaning step during trials. The control PDMS substrate (the 1000 μm substrate) was provided by Sharklet Technologies, Inc (Aurora, CO), and is the same substrate used in Stark *et al*.^9^. All substrates were supported on 0.25 inch thick glass sheets.

Sylgard 184 PDMS (Dow Corning, Midland, MI) substrates of different thicknesses were made using methods specific to the desired thickness. For the 90 μm substrate, a doctor blade (MTI Corporation, Richmond, CA) was used. The PDMS, mixed at a 9:1 ratio^24^, was placed onto a clean (soap/water, IPA, then air dried) glass sheet behind a 150 mm wide doctor blade with the height set using a known reference. Just before applying the PDMS, the glass was cleaned with acetone, dried with a Kimtech wipe, and the dust was blown off using a hand-held air puffer. The PDMS mixture was spread using a motion table, which pulled the glass at a consistent speed but held the doctor blade in place. The 10 μm substrate was made using a 25 wt% solution of the 9:1 PDMS in hexane. This solution was cast onto the prepared glass and pulled with a doctor blade by hand. Both substrates were cured at ambient conditions for at least 48 hours, with an additional curing step for the 10 μm substrate in a 60 °C oven for 24 hours to ensure complete solvent evaporation.

The 2 nm PDMS substrate was made by cleaning the glass thoroughly (soap and water, followed by DI water, ethanol, and acetone before being placed in a strong base bath for 20 minutes, rinsed with DI water, nitrogen dried, and placed in a 120 °C oven), then pouring a 5 wt% solution of Sylgard 184 PDMS in toluene over the glass surface. The glass was then rinsed with pure toluene and placed in a 60 °C oven for 24 hours. Additional small pieces of glass were made following this procedure for use in friction measurements.

The cellulose acetate/foam/glass or cellulose acetate/polyurethane/glass layered composites were prepared by gluing either EVA foam (Creatology, Michaels, Irving, TX) or a polyurethane sheet (McMaster-Carr, Elmhurst, IL) to a glass sheet using a cyanoacrylate glue. Both the foam and the polyurethane sheets as purchased had adhesive backings, which were used to adhere the cellulose acetate sheets (CG5000, 3 M, Maplewood, MN). The cellulose acetate/glass composite was produced by adhering the cellulose acetate directly to the glass using a thin, smooth layer of cyanoacrylate glue. Substrates were allowed to cure for at least 48 hours in ambient conditions before testing.

### Surface Characterization

#### Coating coverage and thickness

Each PDMS substrate was sprayed with water to determine whether the surface was completely covered with PDMS. Any wetting areas would result in the substrate’s rejection. Coating thickness was analyzed post gecko testing by fracturing a 1 inch strip of composite from the middle of the sample, in the gecko testing area. This strip was then broken under liquid nitrogen to allow for brittle fracture of the PDMS. The cross sections were examined at 9 locations using a microscope to determine the thickness of the PDMS layer. For the 2 nm PDMS coating, microscopy was ineffective. Therefore, silicon wafer pieces were coated, following the same procedure as the 2 nm PDMS sheet preparation (with the substitution of plasma cleaning for the base bath) and the coating thickness was analyzed using a J Woolam Ellipsometer (Lincoln, NE), modeling the PDMS as a Cauchy layer.

#### Coating properties

Friction and surface energy of the surface in contact with the geckos were measured. For friction testing, new PDMS lenses and samples were prepared by mixing Sylgard 184 at a 10:1 ratio of base to crosslinker, which is standard procedure for the preparation of our PDMS lenses^36^. Testing of 5:1 and 10:1 Sylgard 184 showed no difference in pull-off force^37^, therefore there should not be significant difference between our 9:1 and 10:1 substrates for whole animal and friction testing, respectively. The samples were made with 10:1 ratio of base to crosslinker as well to maintain a symmetric system in the testing of friction of PDMS substrate with a PDMS lens. After the evacuation of bubbles by a vacuum dessicator, the lenses were formed by extruding hemispheres of the mixture underwater onto a polystyrene petri dish. The PDMS sheets were made by pouring the evacuated mixture into a polystyrene petri dish. The PDMS was cured in an oven for 4 hours at 60 °C. The extraction process for the friction measurements consisted of placing the lenses and sheets into glass petri dishes in toluene for two weeks. The solvent was changed every 48 hours. After the final solvent removal, the covered dish with PDMS was allowed to dry overnight in the hood, followed by vacuum drying at 60 °C for 4 hours and then was kept under vacuum overnight.

The friction was measured using a homebuilt friction cell described elsewhere^36^. Briefly, the lens was taped onto a glass plate, which was taped onto a rigid arm. Then the PDMS substrate was adhered through interaction with another glass plate, which was then taped to an arm movable in the x and y directions. The normal forces and shear forces were measured using capacitor plates attached to spring steel. The substrate was brought into contact with the lens using picometer motors. The shear forces were measured under sliding for the applied normal forces of 5 mN, 10 mN, 20 mN, and 30 mN. The coefficient of friction was calculated using the slope of the line of the average shear force divided by the average normal force in the plateau region for each trial^38^. Each trial was performed on a unique spot, and the lens was changed between each set of four forces.

Static water contact angles were measured post gecko trials using a goniometer (ramé-hart, Succasunna, NJ), with 1.4 μL droplets. Substrates were cleaned as during trials, then measurements were taken in the areas most frequently stepped on by the geckos during shear adhesion trials.

#### Modulus measurements

Both composite modulus and surface modulus (using a nanoindenter) were measured for each substrate, as presented in Table 1. The composite moduli for the Sylgard PDMS substrates (2 nm, 10 μm, and 90 μm) were calculated using a simple series spring model, where the change in each composite thickness, Δ*L_T_*, is the sum of the change in composite layer thicknesses (Δ*L*_1_ and Δ*L*_2_). From Hooke’s Law, we can write the change in layer thickness as a function of the force applied (*F*), the original thickness *L*, the area *A* over which the force acts, and the layer’s elastic modulus *E* (equation (1)). Considering the layers as springs in series, where the force is equally applied to each layer, the cross sectional area and the force on each spring are also equal, allowing us to write the total composite modulus (*E_T_*) as a function of the composite thickness and the layers’ elastic moduli and individual thicknesses (equation (2)).


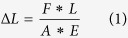



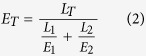


The 1000 μm PDMS substrate and the cellulose acetate substrates contain materials with unknown elastic moduli, therefore the composite moduli were calculated from measured hardness values. Composite hardness was measured using a Shore A durometer, as the materials were thick enough to meet the ASTM standard D 2240-04. Their composite moduli were then calculated from the measured hardness using the theoretical conversion from the durometer value (equation (3)), where *S* is the Shore A value^39^. Equation (3) was developed for rubbers with Shore A hardnesses greater than 40; the cellulose acetate surfaces are composites, rather than rubbers, and therefore the calculated values are not precise. Our substrates range over 5 orders of magnitude in modulus, and therefore the impact of these errors on our conclusions is expected to be minimal.


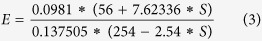


The modulus at the surface of the composites was measured using a TI Premier nanoindenter (Hysitron, Minneapolis, MN). Samples were secured to AFM disk mounts using cyanoacrylate glue, and tested in 4 locations using a low-load Berkovich tip. The glass and 2 nm PDMS substrates were tested using a load controlled standard test, and the cellulose acetate and remaining PDMS substrates were tested using a displacement controlled test, where the materials were indented 1 μm. From the force-displacement curve, reduced modulus was calculated using the instrument’s software.

### Whole Animal Experiments

Whole animal experiments followed the guidelines of the Society for the Study of Amphibians and Reptiles (SSAR 2015) and were approved by the University of Akron IACUC protocol 07-4 G. The trials consisted of 7 Tokay geckos (*Gekko gecko*) from the University of Akron Research Vivarium. The geckos used ranged in weight from 50–132 g. All specimens were housed in a temperature and humidity controlled environment; the room was maintained at a 25 ± 1 °C with a relative humidity of 75–80%. The room was set to a 12 hour photoperiod with UVA/UVB full spectrum lights, simulating the geckos natural circadian rhythm. Heating tape along the underside of each tank allowed the geckos to thermoregulate within the range of 23–25 °C, typical for free-ranging geckos. Each gecko was fed three times a week (vitamin and calcium dusted cockroaches, with a fruit supplement of baby food) and was individually housed in a ten gallon glass tank that was misted daily. Geckos’ toenails were clipped a week prior to experiments.

All trials were done in an environmental chamber, set to 25 ± 0.5 °C and 35 ± 5% RH. The specimens were placed in the environmental chamber thirty minutes prior to experimentation to allow them to acclimate to the conditions. Two sets of substrates (cellulose acetate and PDMS) were used in the experiment, each with a glass backing. Velcro strips were placed on the back of the glass layer to allow each substrate to adhere to the force-sensing apparatus, similar to the one used by Niewiarowski *et al*.^40^. This force-sensing apparatus was used vertically and measured the force that the specimens were capable of withstanding (for safety of the gecko, a maximum of 20 N was set).

Before starting an individual experiment, the gecko had a small strip of medical tape carefully placed around the mouth, avoiding the nostrils, and was fitted with two harnesses around their pelvis, which were then attached to a force sensor positioned vertically on a motorized track. The gecko was placed on the substrate and encouraged to take a few steps, ensuring natural adhesion to the substrate. Both the maximum and the ending forces (when all four feet slipped) were recorded. After each trial the substrate was cleaned with ethanol and reverse osmosis water. The cellulose acetate substrates were then wiped completely dry with a Kimtech wipe, while the PDMS substrates were air dried to avoid fiber transfer from the wipe. Geckos were tested a maximum of three times before having at least a 24 hour rest period.

Within each substrate set, gecko trials were performed randomly on the different modulus substrates, with a total of three trials per substrate. For each gecko, the maximum shear force from these three trials was then considered to be their maximum shear force on that surface. In the vertical orientation, the measured shear force of adhesion is the shear force a gecko is able to support in addition to their own body weight. When the geckos are unable to support their own body weight, and slide down the substrate, the shear force is designated to be 0 N.

### Isolated Lamella Experiments

Skin sheds from 7 naturally molting Tokay geckos were collected and stored in a −20 °C freezer. Single lamellae were isolated from gecko sheds and adhered to small glass plates with cyanoacrylate glue such that the skin was anchored to the glass, but the setal strip was free to flex. Shear adhesion trials were done with ethanol/water cleaned glass and solvent-extracted 1000 μm PDMS, of similar size to the plates supporting the lamella. To test the shear adhesion, the friction cell described earlier was utilized. The substrate was brought in contact with the lamella in the normal force direction by motor and loaded to a force of around 5 mN. The lamella was then loaded in the shear direction with a velocity of 5 μm/s such that the substrate would move towards the direction of the tip of the toe, with respect to the lamella. The trial was ended manually when the shear force dramatically dropped, reached a plateau for at least a minute, or the lamella reached the end of the substrate, and the maximum force was taken to be the shear adhesion force.

### Statistical Analysis

Whole animal shear forces measured on glass before and after exposure to PDMS were analyzed, as were the shear forces obtained on the glass and 1000 μm PDMS substrates (for both whole animal and the isolated lamella experiments). We used a mixed model repeated measures ANOVA to test the significance of substrate type on shear force for each experiment, treating gecko as a random effect. We also analyzed the results using a repeated measures MANOVA and obtained qualitatively similar results. We report the results from the former throughout.

### Data availability

Data generated during this study are available through figshare at https://figshare.com/s/79e5d2a4b30233d8a4d7.

## Additional Information

**How to cite this article:** Klittich, M. R. *et al*. Influence of substrate modulus on gecko adhesion. *Sci. Rep.* **7**, 43647; doi: 10.1038/srep43647 (2017).

**Publisher's note:** Springer Nature remains neutral with regard to jurisdictional claims in published maps and institutional affiliations.

## Figures and Tables

**Table 1:** Contact angle (n = 5), hardness (on Shore A scale, n = 10), calculated composite modulus, and measured surface modulus of each substrate (n = 6).

Substrate	Coating Thickness (*μ*m)	Contact Angle(°)	Shore A Hardness	Composite Modulus (MPa)	Surface Modulus (MPa)
Cellulose acetate/foam	N/A	76 ± 1	77.4 ± 0.2	8.0 ± 0.1	1415 ± 19
Cellulose acetate/polyurethane	N/A	76 ± 1	89.7 ± 0.6	21 ± 1	1660 ± 8
Cellulose acetate/glass	100	76 ± 1	N/A	45120 ± 18	4155 ± 45
1000 μm PDMS	1064 ± 28	115 ± 2	70.9 ± 0.2	5.8 ± 0.1	7 ± 0
90 μm PDMS	89 ± 1	103 ± 1	N/A	109 ± 2	5 ± 0
10 μm PDMS	10 ± 1	110 ± 1	N/A	970 ± 70	24 ± 1
2 nm PDMS	0.002 ± 0.0006	103 ± 1	N/A	69070 ± 200	77040 ± 110

Moduli for cellulose acetate/foam and cellulose acetate/polyurethane were calculated from the Shore A measurements, using equation (3), while moduli for PDMS substrates were calculated using equation (2). Values are means ± standard error.

**Table 2:** Friction coefficients of 2 nm PDMS, solvent extracted, and un-extracted PDMS substrates, measured with an extracted PDMS lens.

Surface	Friction coefficient
2 nm PDMS	2.2 ± 0.1
Extracted PDMS	1.8 ± 0.1
Unextracted PDMS	1.5 ± 0.1

Values are means ± standard error, n = 3.

**Table 3:** Repeated measures fixed effect table for whole animal adhesion to glass before and after exposure to PDMS, as well as whole animal and isolated single lamella shear adhesion on glass and 1000 μm PDMS substrates.

Test	Source	D.F.	D.F. Denominator	F	Prob>F
Glass Whole Animal	Pre/Post PDMS	1	6	0.3705	0.5651
Whole Animal	Substrate	1	6	74.2921	< 0.0001^∗^
Isolated Lamella	Substrate	1	6	68.2301	0.0002^∗^

Statistically significant p values are starred.
