# Erratum: Influence of substrate modulus on gecko adhesion

**DOI:** 10.1038/srep46587

**Published:** 2017-04-24

**Authors:** Mena R. Klittich, Michael C. Wilson, Craig Bernard, Rochelle M. Rodrigo, Austin J. Keith, Peter H. Niewiarowski, Ali Dhinojwala

Scientific Reports 7: Article number: 4364710.1038/srep43647; published online 03 13 2017; updated on 04 24 2017

This Article contains an error in the legend of Figure 2 where the symbols corresponding to calculations using the spring model are incorrectly given as solid shapes. The Figure and accompanying Figure legend appear below.

## Figures and Tables

**Figure 2:**
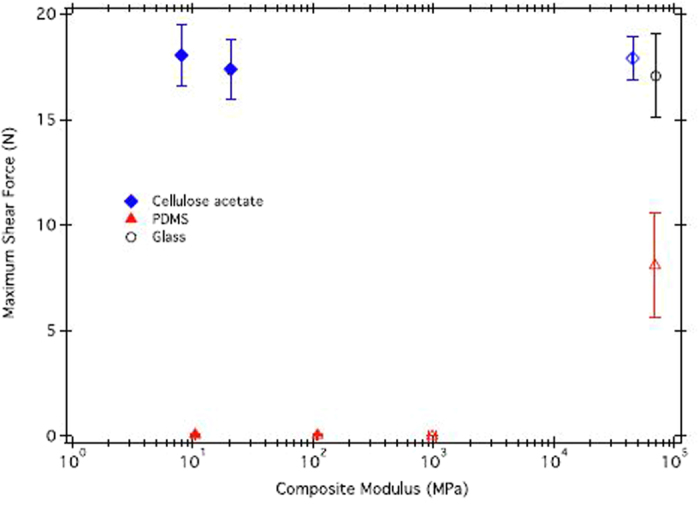
Average maximum shear force of adhesion for whole animals on cellulose acetate (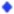) and PDMS (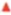) substrates of varying composite moduli. Composite moduli are calculated from the spring model (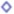 , 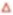) or from durometer measurements (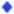, 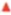), and are tabulated in Table 1. Values are means ± standard error, n = 7.

